# The role of soft-tissue traction forces in bone segment transport for callus distraction: A force measurement cadaver study on eight human femora using a novel intramedullary callus distraction system

**DOI:** 10.1007/s11751-015-0220-8

**Published:** 2015-03-28

**Authors:** Konstantin Horas, Reinhard Schnettler, Gerrit Maier, Gaby Schneider, Uwe Horas

**Affiliations:** 1 Bone Research Program, ANZAC Research Institute, University of Sydney, Gate 3 Hospital Road, Concord, 2139 Australia; 2 Department of Trauma Surgery/Laboratory of Experimental Trauma Surgery, Justus-Liebig-University, Rudolf-Buchheim-Str. 7, 35392 Giessen, Germany; 3 Institute of Mathematics, Goethe-University, Robert-Mayer-Str. 10, 60325 Frankfurt, Germany; 4 Department of Orthopaedic and Trauma Surgery, Kliniken des Main-Taunus-Kreises GmbH, Kronberger Str. 36, 65812 Bad Soden, Germany

**Keywords:** Traction force measurement, Soft tissues, Callus distraction system, Intramedullary, Distraction osteogenesis, Bone defect treatment

## Abstract

Callus distraction using bone segment transport systems is an applied process in the treatment of bone defects. However, complications such as muscle contractures, axial deviation and pin track infections occur in the treatment process using the currently available devices. Since successful treatment is influenced by the applied distraction force, knowledge of the biomechanical properties of the involved soft tissues is essential to improve clinical outcome and treatment strategies. To date, little data on distraction forces and the role of soft-tissue traction forces are available. The aim of this study was to assess traction forces generated by soft tissues during bone segment transport using a novel intramedullary callus distraction system on eight human femora. For traction force measurements, bone segment transport over 60-mm femoral defects was conducted under constant load measurement using 40- and 60-mm bone segments. The required traction forces for 60-mm bone segments were higher than forces for 40-mm bone segments. This study demonstrates that soft tissues are of relevance biomechanically in bone segment transport. The size of the bone segment and the selection of the region for osteotomy are of utmost importance in defining the treatment procedure.

## Introduction

The technique of callus distraction for the treatment of bone defects started at the beginning of the nineteenth century [1]. Despite significant improvements, the treatment of large bone defects remains challenging in many ways. Callus distraction can be accomplished either through extramedullary systems (EMS) such as the Ilizarov method or through totally implantable intramedullary distraction systems (IMS) [2–5]. External fixators in EMS are poorly accepted by patients, frequently resulting in pain, stiffness, irritation and pin track infections [6]. The overall clinical utility for EMS is poor [7]. Even though IMS avoid the problem of pin track infections and are preferred in maintaining quality of life, they are not widely used as the existing models are still limited in terms of function and control [8]. It is known that a series of biological and mechanical elements, most of them not fully understood, are involved in the distraction process. Essential information on variables such as the best velocity of distraction, the traction forces involved in the transport process and whether or not the position of the osteotomy is of relevance is lacking [9].

In order to improve on existing models and develop a novel callus distraction systems (CDS), basic biomechanical knowledge regarding traction forces involved in bone segment transport (BST) is needed. A better understanding may help reduce frequent complications such as muscle contractures, axial deviation and traction injury to vessels and nerves but would also allow modifying the treatment regimen. There are little data on forces in callus distraction systems. Previous studies measured distraction forces either in complicated or in using inaccurate systems ignoring frictional force; many were conducted in animal experiments having less relevance for human callus distraction [10–14]. Whether the predominant part of the force is generated by the viscoelasticity of the soft tissues or by the callus itself, is uncertain.

The overall force required for BST consists of several different load components [9, 15, 16]. All adherent structures of the transporting bone segment such as tendons, fasciae and muscles generate a force due to the distraction of the soft tissues. Another component of the overall traction force is directly related to the callus and the new forming bone tissue of the regenerate. The tissue reproduction in callus distraction is stimulated by traction, and the process is known as distraction osteogenesis [17]. A percentage of the force can be attributed to the displacement of the tissue blocking the bone defect and some is directly related to the measuring system itself. Precise knowledge on the applied forces involved in the treatment process is fundamental. The data are diverse in the literature on the overall force required for callus distraction but also the distribution of forces. This study aims to measure the mechanical forces applied by the soft tissues in BST over the whole period of distraction using a novel intramedullary CDS.

## Materials and methods

Bone segment transport (BST) over a 60-mm femoral defect was conducted on eight human femora (four cadavers) under constant load measurement. For that purpose, a 40-mm bone segment on the right femur and a 60-mm bone segment on the left femur were generated on each cadaver. All cadavers were frozen to a temperature of −18° Celsius exactly 48 h after *exitus letalis* and defrosted for a period of 24 h prior to the experiment. Each cadaver was carefully selected, and none had a history of bone injury or any musculoskeletal disease that could have had an impact on the measurement.

**Fig. 1:**
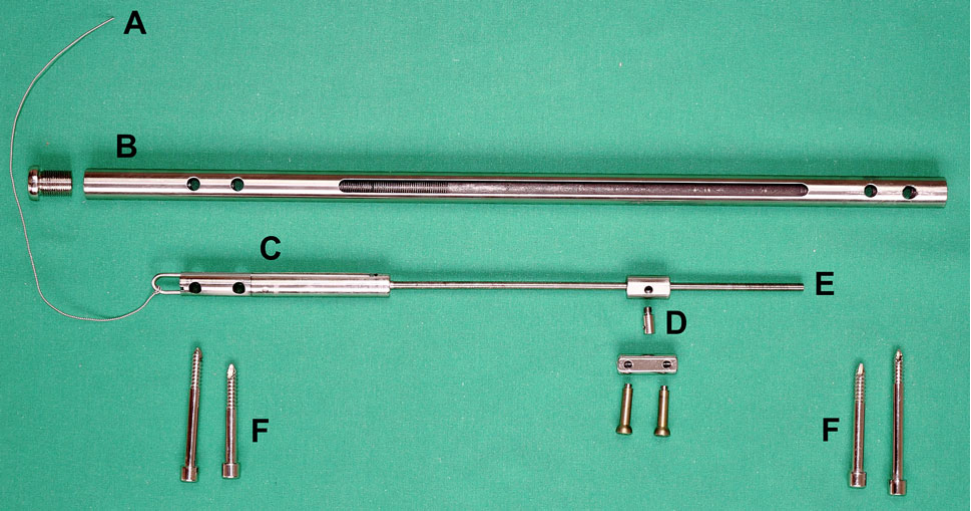
Individual components of the CDS: *A* traction wire, *B* nail, *C* mechanics, *D* threaded rod spindle, *E* threaded rod, *F* interlocking screw

**Fig. 2:**
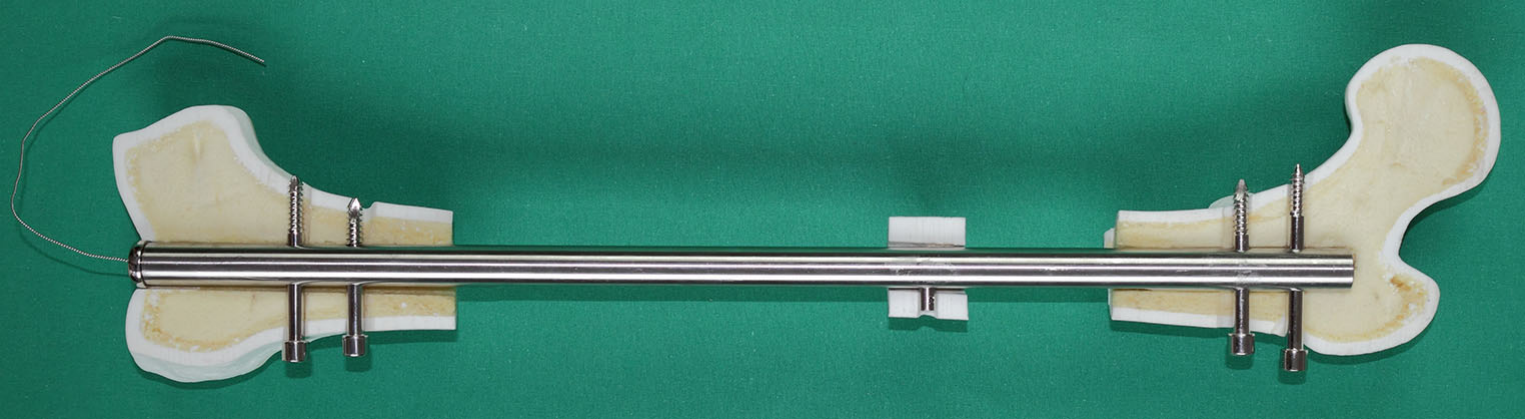
CDS implanted into the femur (anteroposterior view): the traction wire is connected to the fully inserted mechanics

**Fig. 3:**
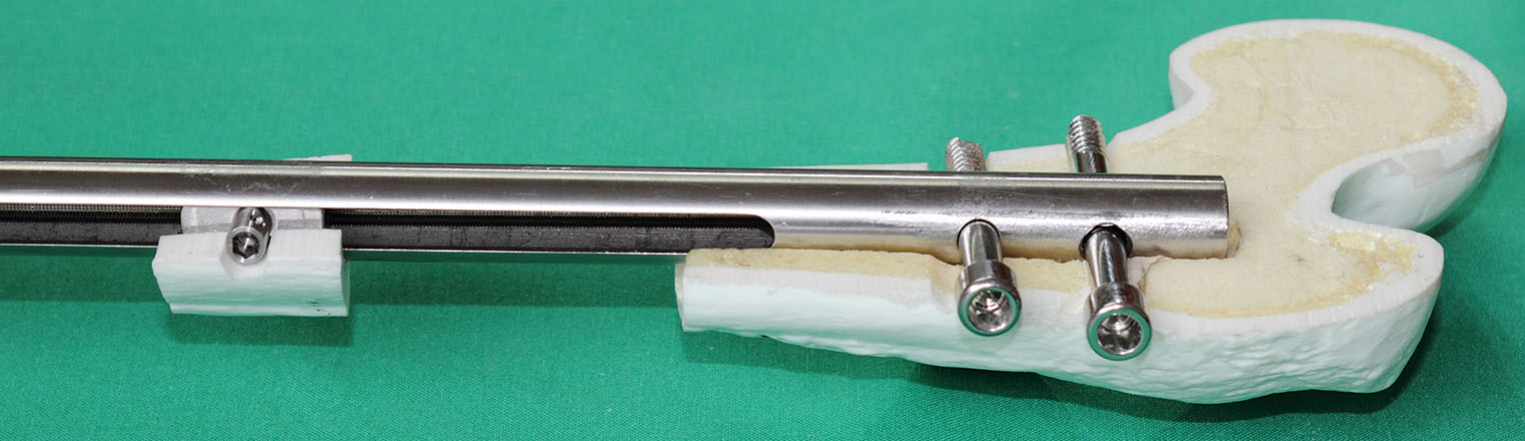
CDS implanted into the femur with bone segment connected to the threaded rod (lateral view)

**Fig. 4:**
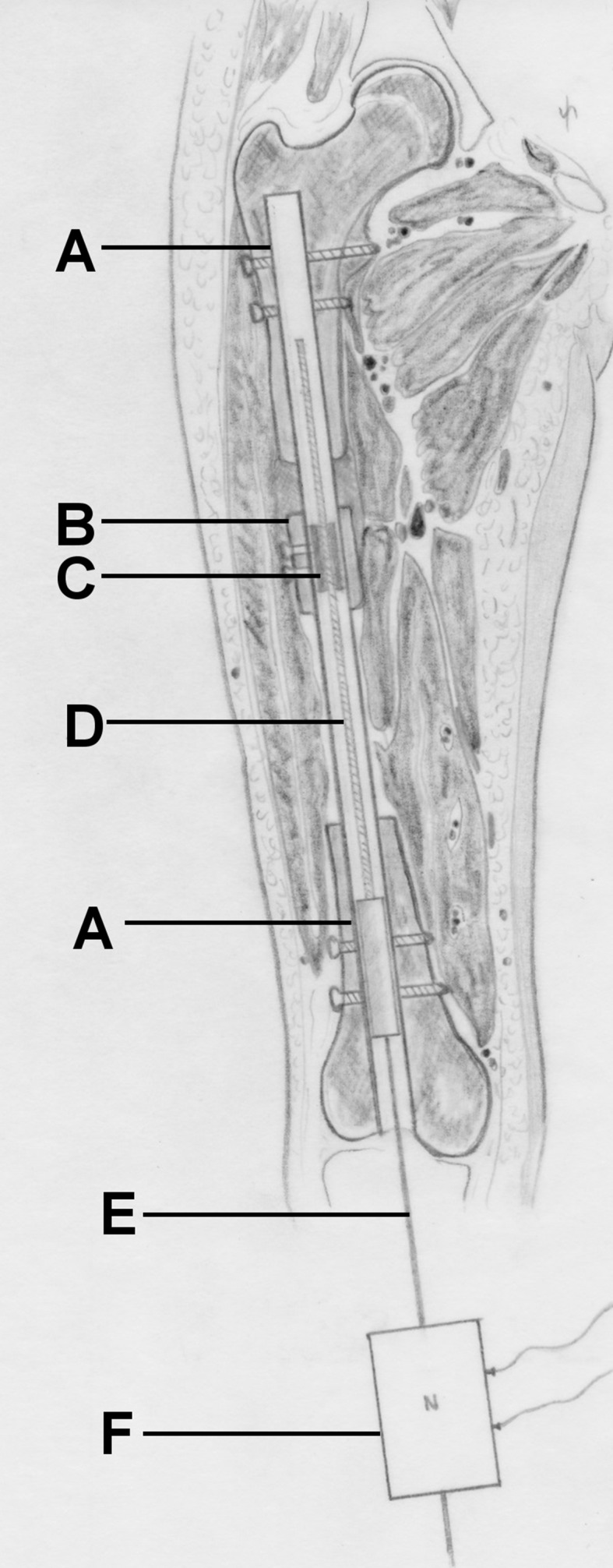
Schematic of the CDS implanted into the femur. *A* nail, *B* bone segment, *C* threaded rod spindle, *D* threaded rod, *E* traction wire, *F* load cell. By pulling on the load cell connected to the distal wire, bone segment transport is conducted and the required traction force can be measured simultaneously

The novel CDS used in this experiment comprise an intramedullary nail with transverse interlocking screws and an in-line mechanism, with a threaded rod and a threaded rod spindle sitting on top, which produces the connection between the bone segment and the threaded rod (Fig. 1) [18]. A longitudinally slit interlocking nail keeps the defective area open throughout distraction (Figs. 2, 3). The bone segment is moved in the direction of the desired callus distraction by rotating the threaded rod in a specific way. This rotation is achieved by the mechanism converting a traction force produced by a connected wire into a translational movement. In order to move the wire and trigger the mechanics for BST, a traction force is generated by pulling on the connected wire with a load cell being interposed to measure the generated amount of force (Fig. 4). For clinical application, the traction wire was designed to be fixed to the tibial tubercle, and BST is conducted by generating a traction force on the wire by flexion of the knee joint.

### Implantation

In order to avoid damage to the surrounding tissues of the femur, a medial approach was chosen to generate the 60-mm bone defects. The CDS were implanted into the femur via a standard retrograde transarticular nailing in the supine position. Proximal locking of the CDS was carried out in an anterior/posterior direction in the peritrochanteric region of the femur and distal transverse locking in a lateral to medial direction. Bone segments for transport were produced via a minimal lateral approach and osteotomy directly distal to the insertion of adductor brevis muscle at the linea aspera in such a way as to preserve as much of the periosteum as possible. Each bone segment was then connected to the intramedullary CDS using two transcortical screws.

### Measurement

For calibration and measurement of the traction force, a servohydraulic testing machine (PSA 40KN Schenk, Germany) and a load cell (PCE MA001, Germany) for an effective range up to 1000 Newton were used. For data record and physical checks, BMLab 200 V.2. software was utilised. Prior to traction force measurement, forces generated by the CDS itself were measured using the load cell. These frictional forces were then subtracted from the measured loads further in the experiment. For traction force measurement, the CDS were released every 15 s, leading to a transport distance of 0.25 mm per release. Records of the measurements were made every 2 mm of transport distance from the beginning of distraction to closing at the docking site. For inspection and validation of the running system and the transporting bone segment, radiographs (AP and lateral) were taken throughout the experiment using an X-ray C-Arm (Siemens, Germany).

## Results

**Fig. 5:**
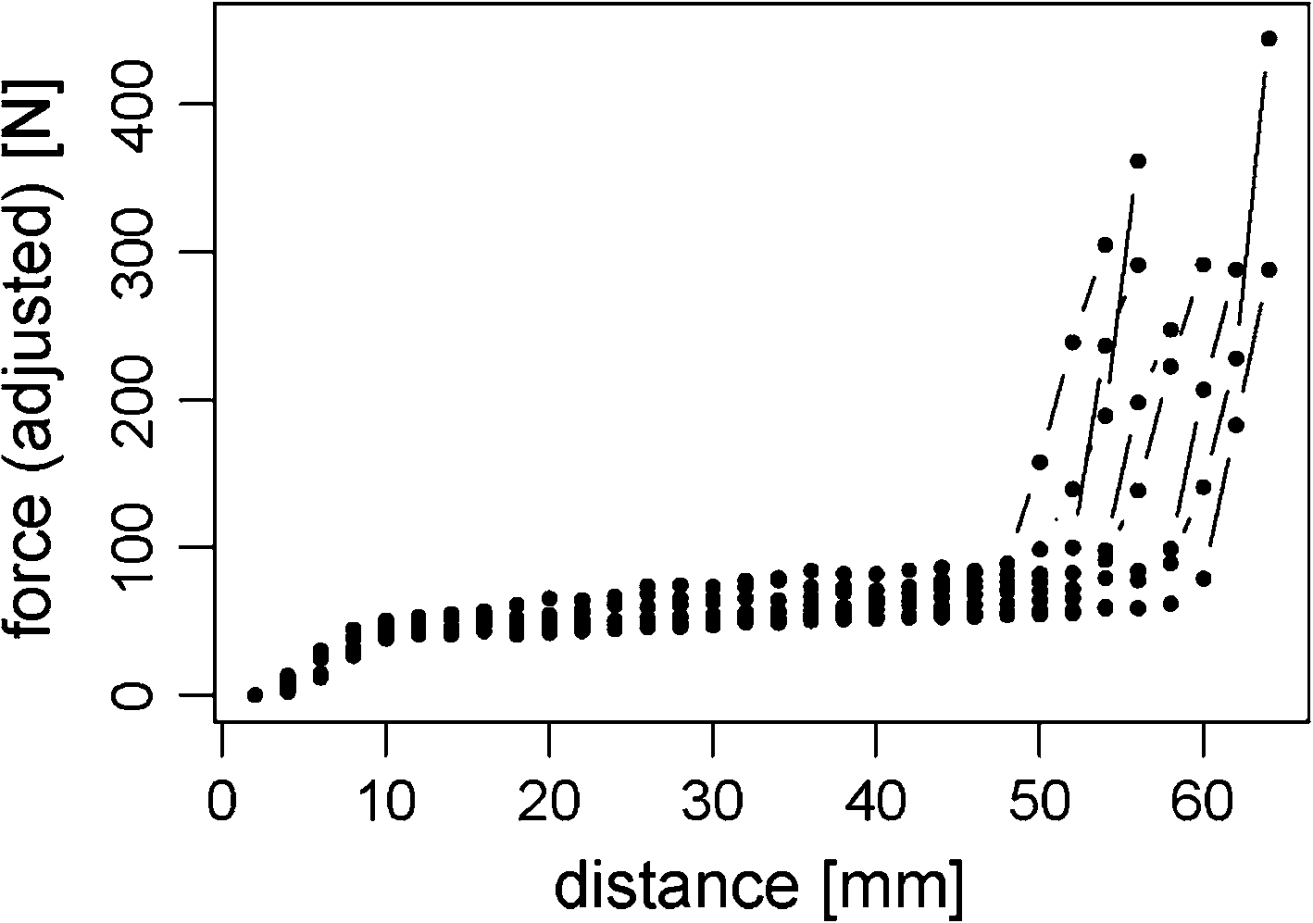
Adjusted force measurement for 40- and 60-mm bone segment transport in eight human femora. *Each curve* represents one bone segment to be transported within one femur

All bone segments were transported to the docking site without any complications. During continuous force recordings and radiographic validation, no mechanical obstacles of the system were observed. The total amount of measurements on each of the eight femora varied from 27 to 32 measurements accounting for a transport distance of 53–64 mm. Traction forces showed the same curve progression and the same force pattern in all of the eight femora (Fig. 5). The recorded force data in the graph can be divided into three different groups. After a short period (0–10 mm transport distance) of relatively steep increase in force, traction force increased roughly linearly with distance (10–50 mm). At 50–60 mm transport distance, the recordings showed a rapid increase in forces up to a maximum of 444.5 N.

**Fig. 6:**
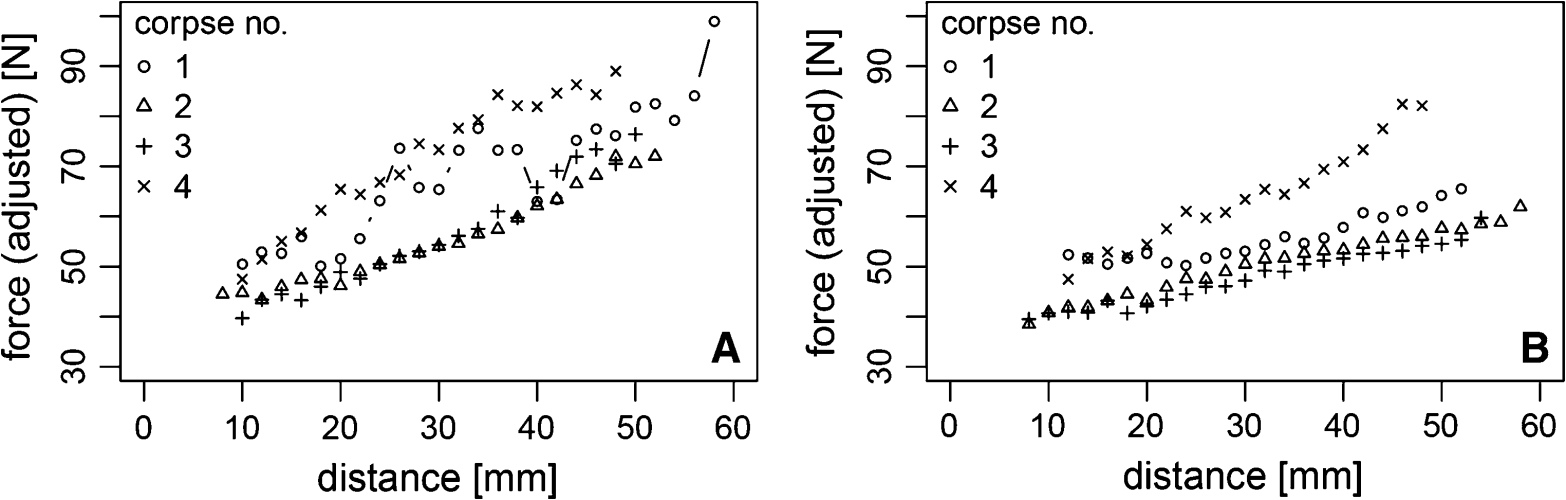
Adjusted force measurement for bone segment transport in four human cadavers using a 60-mm bone segment (**a**) and a 40-mm bone segment (**b**)

For further analysis, we focused on the intermediate part of the graph in Fig. 5 in which the force increased linearly with distance and compared the different bone fragment sizes (Fig. 6). We used simple linear regression in order to describe the parameters of the linear increase in force. The resulting estimates of slope and of mean force at two exemplary values of 20 and 40 mm transport distance are given in Table 1. The estimated slope and the mean force required at 20 mm and 40 mm transport distance were larger in 60 mm than in 40 mm bone segments.

**Table 1:** Summary of ordinary linear regression of the data given in Fig. 5

Cadaver	Slope (N/mm)		Mean force (N) estimated from linear regression			
			At 20 mm transport distance		At 40 mm transport distance	
	60 mm	40 mm	60 mm	40 mm	60 mm	40 mm
1	0.73	0.35	57.9	51.5	73.4	58.5
2	0.68	0.43	48.9	45.0	62.5	53.4
3	0.88	0.39	47.6	43.5	65.2	51.4
4	1.01	0.89	62.3	54.9	83.2	72.8

For each cadaver, the slope of the regression line and the estimated value at 20 and 40 mm transport distance are given. The estimated slope and the mean force required at 20 and 40 mm transport distance were larger in 60 than in 40 mm bone fragments

Due to the small number of cadaveric specimens, we omitted statistical tests of significance. However, we investigated potential relationships between the slope of the regression and several demographic variables. No clear relation of age, weight and body mass index to traction force was visible.

## Discussion

Leong et al. [15] were the first to report distraction loads in humans using an EMS consisting of Steinmann pins and a distraction frame in 1979. In this study, the authors described a time-dependent viscoelastic behaviour of stretched tissues during limb lengthening (LL). Although Aronson et al. agreed that an increase in distraction loads might be due to elastic tissue resistance, they hypothesised that a large portion of the distraction load was generated by the callus itself [10]. This was supported by a study by Younger et al. [12] investigating femoral forces during LL in children. They suggested that the presence of an inelastic callus in LL mainly affects the magnitude and increase in force. In contrast, Wolfson et al. [19] indicated that traction forces are generated by the soft tissues of the leg from passive stretch or muscular activity. Simpson et al. [11] supported this theory in their studies on LL comparing forces in patients with post-traumatic shortening to patients with congenitally short limbs. They found a noticeable difference in force between these two groups. In particular, patients with congenitally short limbs developed higher peak forces due to the resistance of the soft tissues. Also known, as indicated by Forriol et al. [14], is that callus properties do not vary substantially while LL is performed. In their opinion, the behaviour in force pattern is likely due to the mechanical properties of the soft tissues. Contrastingly, Brunner et al. [13] suggested that the percentage of muscle and soft-tissue forces of the total force measured in their study on BST in sheep was very low.

There are two existing theories for the generation of the main forces measured in callus distraction: some authors suggest that bone segment transport forces cannot be attributed to the soft tissues, whereas others believe that the soft tissues play a decisive role in force generation. The present study indicates that soft tissues are of relevance in BST biomechanically. As this study was performed on human cadavers, no callus formation was possible, and therefore, no callus-related force was included in the measurements. In contrast to the studies mentioned above, frictional forces of the distraction system were measured prior to the experiment and were subtracted from the resulting data later in the experiment leaving the final force data representing that solely generated by all adherent structures of the bone segment and by the tissues blocking the bone gap. The three-part curve progression shown in the results is interpreted as follows: the initial increase in force can be related to soft-tissue tension from implantation of the CDS (0–10 mm); the subsequent almost linear increase with distance represents the distraction force generated by the soft tissues (10–50 mm); and the rapid increase in force at the end of the transport distance can be related to the soft tissues blocking the bone gap and the impact of the bone segment at the docking site. Forces for 60-mm bone segments were higher throughout the whole period of BST, indicating that a higher amount of adherent tissues had been involved in the transport process (Fig. 6).

As with any cadaveric test set-up, this study has some limitations. Measurements in cadaveric specimens eliminate active muscle forces, which limits the documentation of generated forces to an observation of passive forces solely. As the CDS were released every 15 s to generate a transport distance of 0.25 mm per release until impact of the bone segment at the docking site, this would contrast to the clinical situation where the speed of segment transport is around 1 mm per day allowing some time for the soft tissues to relax between transport intervals. As the soft tissues in our experimental set-up were given 15 s to adapt after each release, it is likely that the viscoelasticity of the soft tissues maintains some resistant force that falsely increases the total amount of force measured. Despite these limitations, we believe that these findings are of value for clinicians designing the treatment regimen. The study shows that transport forces for 60-mm bone segments were higher than transport forces for 40-mm segments, which will have less adherent soft tissues. For this reason, not only the size but the site of the bone segment has to be taken into consideration while defining the treatment procedure.

This is the first described force measurements using an IMS, and there are no previous studies for comparisons to be made. In a similar study presented by Baumgart et al. [16], the force curve progression demonstrated the same pattern of behaviour as in the current authors’ subjects. However, as there are no data available on traction forces for BST in living humans measured in IMS, no exact conclusion of the amount of force generated by the soft tissues contributing to the overall force is possible. Despite this, the data above suggest that forces generated by the soft tissues contribute to the overall forces involved in BST.

## Conclusion

This study is a first approximation of a force measurement in human cadavers using an IMS. We showed that soft tissues are of relevance in BST and for this reason the amount of force required for BST depends on the size and the localisation of the bone segment to be transported. The findings of this study are of value for the improvement of currently available CDS and might contribute to a reduction in complications in the treatment of bone defects.
